# Supplementary material for: Identification of photoperiod-induced specific miRNAs in the adrenal glands of Sunite sheep (Ovis aries)
Source: Front Vet Sci. 2022 Jul 22;9:888207. doi: 10.3389/fvets.2022.888207 (PMC9354845; doi:10.3389/fvets.2022.888207)
Supplement: Supplementary file 1 [file Data_Sheet_1.ZIP › Supplementary file/miRDeep2 file.zip!/miRDeep2 file/Know_Structure/SP42A1/oar-mir-543.pdf]

miRBase precursor : oar-mir-543
Total read count : 8950
oar-miR-543-5p read count : 5
oar-miR-543-3p read count : 8916
remaining reads : 29

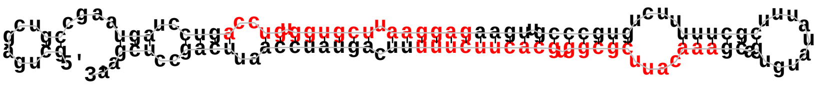

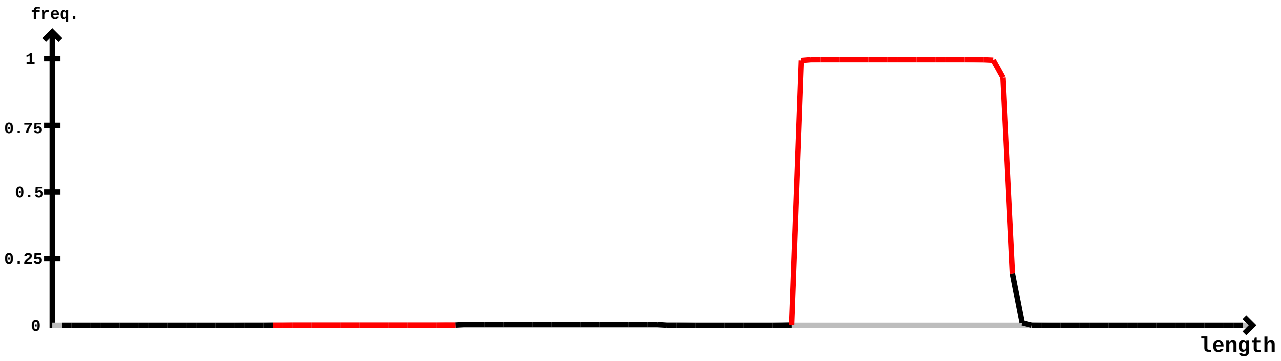

oar-miR-543-3p

oar-miR-543-5p

```
5'- gcugagcugccgaaugauccugaccuguggugcuuaaggagaaguugcccgugucuuuuucgcuuuauaugugacgaaacauucgcggugcacuucuuuuucaguauccaauucagccucgaa  -3'   exp
    ((......)).....((..((((..((.((((((.((((((((((.(((((((....((((((.........).)))))....))))).))))))))))))).))))))))..))))..))...   reads   mm
    ..................ccugaccuguggugcuuaagga...................................................................................   1       0
    .....................gaccuguggugcuuaagga...................................................................................   1       0
    .....................gaccuguggugcuuaaggag..................................................................................   2       0
    .......................ccuguggugcuuaagga...................................................................................   2       0
    .......................................agaaguugcccgugucuuuu................................................................   1       0
    .......................................agaaguugcccgugucuuuuucg.............................................................   1       0
    ........................................gaaguugcccgugucuuuuucg.............................................................   3       0
    .........................................aaguugcccgugucuuuuuc..............................................................   2       0
    .........................................aaguugcccgugucuuuuucg.............................................................   18      0
    .........................................aaguugcccgugucuuuuucgcu...........................................................   1       0
    .........................................aaguugcccgugucuuuuucgcuu..........................................................   2       0
    ..........................................................................cgaaacauucgcggugcacuucu..........................   3       0
    ...........................................................................gaaacauucgcggugcacuucu..........................   5       0
    ...........................................................................gaaacauucgcggugcacuucuu.........................   1       0
    ............................................................................aaacauucgcggugcacu.............................   1       0
    ............................................................................aaacauucgcggugcacuu............................   3       0
    ............................................................................aaacauucgcggugcacuuc...........................   14      0
    ............................................................................aaacauucgcggugcacuucu..........................   573     0
    ............................................................................aaacauucgcggugcacuucuu.........................   6563    0
    ............................................................................aaacauucgcggugcacuucuuu........................   1643    0
    ............................................................................aaacauucgcggugcacuucuuuu.......................   80      0
    ............................................................................aaacauucgcggugcacuucuuuuu......................   2       0
    ............................................................................aaacauucgcggugcacuucuuuuuc.....................   1       0
    .............................................................................aacauucgcggugcacuucu..........................   1       0
    .............................................................................aacauucgcggugcacuucuu.........................   17      0
    .............................................................................aacauucgcggugcacuucuuu........................   8       0
    ..............................................................................acauucgcggugcacuucuu.........................   1       0
```
